# Comparing the Prognosis of Non‐Small Cell Lung Cancer Patients Who Did Not Undergo Surgery After Neoadjuvant Chemotherapy Combined With Immunotherapy: A Retrospective Study

**DOI:** 10.1111/1759-7714.70280

**Published:** 2026-04-10

**Authors:** Zhenghui Ma, Yuqi Wu, Guangqian Ji, Zongmei Zhou, Xin Wang, Jianyang Wang, Wenyang Liu, Lei Deng, Wenqing Wang, Junlin Yi, Nan Bi, Tao Zhang

**Affiliations:** ^1^ Department of Radiation Oncology, National Cancer Center/National Clinical Research Center for Cancer/Cancer Hospital Chinese Academy of Medical Sciences and Peking Union Medical College Beijing China

**Keywords:** neoadjuvant chemotherapy combined with immunotherapy, nonsmall cell lung cancer, positron emission tomography/computed tomography, radiotherapy, survival and prognosis

## Abstract

**Objectives:**

To evaluate the prognosis of patients with nonsmall cell lung cancer (NSCLC) who did not undergo surgery after neoadjuvant chemotherapy combined with immunotherapy (NACI). Patients were grouped according to subsequent treatment: radiotherapy (RT) or nonradiotherapy (non‐RT), and the prognostic importance of positron emission tomography/computed tomography (PET/CT) was further assessed.

**Materials and Methods:**

This retrospective study included NSCLC patients who received NACI between November 2020 and September 2024 at the Cancer Hospital, Chinese Academy of Medical Sciences, and Peking Union Medical College. Not all patients underwent surgery and subsequently received either RT or non‐RT treatment. Patients were stratified by whether PET/CT was performed and by their SUV‐max values before RT or non‐RT. Progression‐free survival (PFS) and overall survival (OS) were calculated from the start of neoadjuvant therapy using the Kaplan–Meier method.

**Results:**

A total of 73 eligible patients were enrolled: 21 (28.8%) in the non‐RT group (nRTG) and 52 (71.2%) in the RT group (RTG). The median follow‐up time for all patients in the group was 18 months. The results show no significant difference in PFS (*p* = 0.653) or OS (*p* = 0.742) between RTG and nRTG. Among the patients who did not undergo PET/CT examination, the results showed a significant difference in PFS (*p* = 0.022), but no difference in OS (*p* = 0.320). Among the patients undergoing PET/CT examinations, in terms of PFS, compared to nRTG + SUV‐max ≤ 4 g/mL, there is no significant difference in RTG + SUV‐max ≤ 4 g/mL (p = 0.584), and RTG + SUV‐max > 4, < 8 g/mL (*p* = 0.156). However, RTG + SUV‐max ≥ 8 shows a statistical difference (*p* = 0.005).

**Conclusion:**

Among NSCLC patients who did not undergo surgery following NACI, no significant differences in OS or PFS were observed between the RTG and nRTG groups. For patients who did not receive PET/CT evaluation, radiotherapy remains a key therapeutic option. In patients with a PET/CT SUVmax ≤ 4, radiotherapy may be safely omitted. In comparison, patients with a PET/CT SUVmax > 4 should be managed with a comprehensive treatment strategy that includes radiotherapy as the main component. PET/CT plays a critical role in guiding subsequent treatment selection.

## Graphical abstract

This study retrospectively investigated the prognosis of NSCLC patients who did not undergo surgery after NACI. The patients were grouped based on their subsequent treatment: RTG or non‐RTG, and the importance of PET/CT in predicting prognosis was further evaluated.

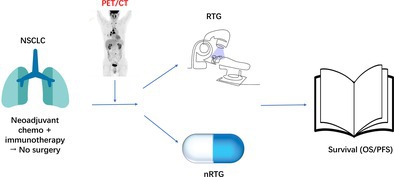

## 1 Introduction

Nonsmall cell lung cancer (NSCLC) accounts for approximately 80%–85% of all lung cancer cases. Surgical resection remains a cornerstone of treatment for patients with resectable disease. Data from the International Association for the Study of Lung Cancer (IASLC) indicate that 25%–70% of patients experience disease recurrence following surgery [1], with 5‐year survival rates declining from 71% in stage IB to 36% in stage IIIA disease [2]. Current guidelines recommend adjuvant chemotherapy for patients with Stage II–III NSCLC after surgical resection, which confers an absolute 5‐year survival benefit of approximately 4%–5% [3].

A meta‐analysis has shown that platinum‐based neoadjuvant chemotherapy significantly improves survival in patients with Stage IB–IIIa NSCLC. By reducing tumor burden, neoadjuvant therapy can increase the likelihood of complete surgical resection. However, compared with surgery alone, preoperative chemotherapy provides only a modest improvement, increasing 5‐year overall survival by 5% and 5‐year recurrence‐free survival by 6% [4].

In recent years, multiple Phase III clinical trials have investigated neoadjuvant immunotherapy combined with platinum‐based chemotherapy. The KEYNOTE‐671 trial demonstrated that neoadjuvant chemotherapy plus pembrolizumab, followed by pembrolizumab maintenance, significantly improved event‐free survival in patients with Stage II–IIIb NSCLC compared with chemotherapy alone (HR, 0.58; 95% CI, 0.46–0.72) [5]. Similarly, the AEGEAN study reported that durvalumab combined with chemotherapy significantly improved event‐free survival (HR, 0.68; 95% CI, 0.53–0.88) and pathological complete response (pCR) rates relative to chemotherapy alone [6]. Findings from CheckMate 816 [7], CheckMate‐77T [8], Neotorch [9], and RATIONAL‐315 [10] further support neoadjuvant chemoimmunotherapy as the preferred treatment strategy for patients with resectable Stage II–III NSCLC. Approximately 20% of patients receiving neoadjuvant chemotherapy combined with immunotherapy (NACI) in these trials did not ultimately undergo surgery due to treatment‐related toxicity, disease progression, or concerns regarding surgical risk. Optimal subsequent management for this subset of patients remains controversial.

Positron emission tomography/computed tomography (PET/CT) is a noninvasive imaging modality that plays an essential role in tumor diagnosis, treatment planning, and response assessment. In lung cancer, PET/CT is widely used to distinguish benign from malignant lesions in the mediastinum and pulmonary parenchyma [11]. PET/CT‐based response evaluation has relied on comparative assessments of FDG uptake before and after treatment. However, in clinical practice in China, relatively few patients undergo baseline PET/CT before therapy. Instead, PET/CT is often performed after NACI or before radiotherapy to evaluate treatment response and assist in radiotherapy target delineation. In such cases, only a single posttreatment PET/CT scan is available, rendering conventional response assessment criteria based on paired imaging inapplicable [12].

This study aims to evaluate the impact of subsequent treatment strategies, radiotherapy versus non‐radiotherapy, on survival outcomes in NSCLC patients who did not undergo surgery following NACI. Further, patients were stratified by postneoadjuvant PET/CT use to assess its value in guiding individualized treatment decisions.

## 2 Methods

### 2.1 Patient Selection and Data Collection

Patients with NSCLC who did not undergo surgical resection following NACI were enrolled from the Cancer Hospital, Chinese Academy of Medical Sciences, and Peking Union Medical College between November 2020 and August 2024. Inclusion criteria were: (1) age ≥ 18 years; (2) cytologically or histologically confirmed NSCLC; and (3) receipt of NACI without subsequent surgery. Exclusion criteria included: (1) history of any prior cancer‐specific treatment; and (2) incomplete clinical information. Clinical and treatment data, including sex, age, clinical stage, smoking history, histological subtype, PD‐L1 expression, radiotherapy dose, PET/CT data, and disease course, were retrieved from the electronic medical records. TNM staging followed the 8th edition of the AJCC classification.

### 2.2 Treatment Regimen

All enrolled patients received PD‐(L)1 inhibitor therapy, either as monotherapy or in combination with chemotherapy. Neoadjuvant treatment was generally administered for 2–6 cycles. The PD‐(L)1 inhibitors used included pembrolizumab, tislelizumab, and other approved agents. Specific treatment regimens were selected in accordance with the Chinese Society of Clinical Oncology (CSCO) or National Comprehensive Cancer Network (NCCN) guidelines.

All patients undergoing radiotherapy received volumetric‐modulated arc therapy (VMAT), with total radiation doses primarily ranging from 50 to 60.2 Gy. Most patients received radiotherapy alone, while a minority received concurrent chemotherapy, most commonly vinorelbine or paclitaxel liposome. Dose constraints for the lungs and other adjacent organs at risk (OARs) were applied in strict accordance with NCCN guidelines.

### 2.3 Study Outcomes

PFS was the time from the first cycle of NACI to disease progression or death from any cause. OS was the time from the start of NACI to death from any cause. Follow‐up continued until April 30, 2025, via text messaging, phone calls, or WeChat. Tumor response was assessed using the Response Evaluation Criteria in Solid Tumors (RECIST) version 1.1. Treatment‐related adverse events (TRAEs) were graded based on the Common Terminology Criteria for Adverse Events (CTCAE) version 5.0.

### 2.4 Statistical Analysis

Chi‐squared or Fisher's exact test was employed for comparisons of nominal categorical variables. The Wilcoxon rank‐sum test was applied to ordinal categorical variables. Kaplan–Meier survival curves for PFS and OS were generated and compared using the log‐rank test. A *p* value < 0.05 was considered statistically significant. Analyses and figures were generated using SPSS version 22.0 (IBM, USA) and GraphPad Prism version 8.0 (Dotmatics, USA). Univariate analysis (UVA) and multivariate analysis (MVA) were performed while employing the Cox proportional hazards regression model.

## 3 Results

### 3.1 Baseline Characteristics

**FIGURE 1:**
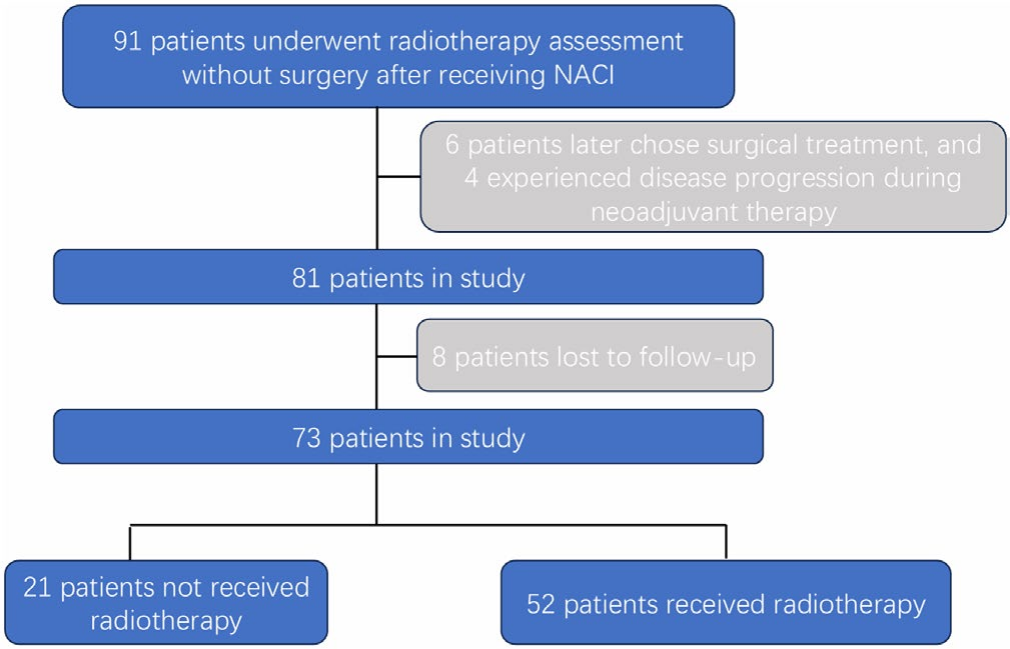
Patient selection flowchart.

A total of 91 patients underwent radiotherapy assessment without surgery after receiving NACI. Of these, six patients later chose surgical treatment, and four experienced disease progression during neoadjuvant therapy (one developed distant metastasis and three showed locoregional progression and received alternative treatments). Moreover, eight were lost to follow‐up. Ultimately, 73 patients were included in the study. Based on treatment decisions following neoadjuvant therapy, patients were assigned to the RT group (RTG; 52 cases) or the non‐RT group (nRTG; 21 cases). Figure 1 shows the flowchart of the patient's selection process.

In the nRTG, reasons for not receiving radiotherapy were: (a) favorable response on PET/CT or CT after neoadjuvant therapy and concern about radiation pneumonitis (*n* = 10, 47.6%); (b) history of pneumonitis or tuberculosis (*n* = 2, 9.5%); and (c) refusal of radiotherapy for other reasons (*n* = 9, 42.9%).

Baseline characteristics are presented in Table 1. Most patients were male, had an ECOG performance status of 0–1, were classified as Stage III, had a history of smoking, and were diagnosed with squamous cell carcinoma. In the nRTG, most patients showed a partial response (PR) on CT or a posttreatment PET/CT SUV‐max ≤ 4 after neoadjuvant therapy and continued with either combined chemotherapy and immunotherapy or maintenance immunotherapy alone. In the RTG, most patients received radiotherapy at a dose ≥ 60 Gy, and most showed PR following NACI. Patients in both groups who underwent PET/CT after NACI were further analyzed and stratified into three subgroups based on SUV‐max values: ≤ 4, 4–8, and ≥ 8 (Table 2).

**TABLE 1:** Baseline demographic and clinical characteristics of the patients.

		RTG (*n* = 52)	nRTG (*n* = 21)	*p*
Age	Median	62.5 (43, 75)	67 (52, 77)	—
Gender	Female	6 (11.1%)	1 (4.8%)	0.373
	Male	46 (88.9%)	20 (95.2%)	
Histology	Non‐SCC	9 (17.3%)	4 (19.0%)	0.860
	SCC	43 (82.7%)	17 (81.0%)	
PD‐L1 status	≥ 1%	24 (46.2%)	8 (38.1%)	0.530
	< 1% + NA	28 (53.8%)	13 (61.9%)	
Smoker	Yes	44 (84.6%)	17 (81.0%)	0.930
	No	6 (11.5%)	3 (14.3%)	
	NA	2 (3.8%)	1 (4.8%)	
*T*	T1	2 (3.8%)	0 (0.0%)	0.426
	T2	22 (42.3%)	13 (61.9%)	
	T3	13 (25%)	4 (19.0%)	
	T4	15 (28.8%)	4 (19.0%)	
N	N0	16 (30.8%)	12 (57.1%)	0.187
	N1	11 (21.2%)	2 (9.5%)	
	N2	23 (44.2%)	6 (28.6%)	
	N3	2 (3.8%)	1 (4.8%)	
Stage	I + II	15 (28.8%)	10 (47.6%)	0.126
	III	37 (71.2%)	11 (52.4%)	
RT dose	< 54	4 (7.7%)	—	—
	≥ 54, < 60	11 (21.2%)	—	
	≥ 60	37 (71.2%)	—	
CT evaluate	CR	2 (3.8%)	2 (9.5%)	0.296
	PR	38 (73.1%)	17 (81.0%)	
	SD	12 (23.1%)	2 (9.5%)	
PET/CT	Yes	24 (46.2%)	11 (52.4%)	0.696
	No	28 (53.8%)	10 (47.6%)	

**TABLE 2:** The SUV‐max in PET/CT after NACI of the patients.

SUV‐max (g/mL)	RTG (*n* = 24)	nRTG (*n* = 11)
≤ 4	5	11
> 4, < 8	8	0
≥ 8	11	0

### 3.2 Efficacy

Survival outcomes were analyzed from the initiation date of NACI, with the data cut‐off set as April 30, 2025. The median follow‐up time for all patients in the group was 18 months. Among all patients, the PFS rates at 1‐ and 2‐years for the nRTG and RTG were 67.7% versus 78.2% and 60.2% versus 22.0%, respectively (*p* = 0.653). The corresponding 1‐ and 2‐year OS rates were 93.8% versus 98.0% and 93.8% versus 80.7%, respectively (*p* = 0.742). UVA and MVA confirmed that squamous cell carcinoma pathology was an independent factor affecting PFS (Table 3).

**TABLE 3:** Univariate and multivariate Cox regression analyses for PFS.

Variables		Univariate			Multivariate		
		HR	95% CI	*p*	HR	95% CI	*p*
Age	< 60 versus ≥ 60	0.698	0.304–1.602	0.397			
Gender	Female versus male	0.524	0.180–1.525	0.236			
Smoker	No versus yes	0.917	0.375–2.242	0.849			
Histology	Non‐SCC versus SCC	0.380	0.174–0.832	0.015	0.344	0.150–0.793	0.012
Stage	I + II versus III	1.632	0.716–3.723	0.244			
PD‐L1	NA + < 1% versus ≥ 1%	1.044	0.597–1.826	0.879			
RT	No versus yes	1.275	0.534–3.047	0.584	1.389	0.572–3.369	0.468
PET/CT	No versus yes	1.113	0.522–2.373	0.782	0.779	0.342–1.777	0.554

**FIGURE 2:**
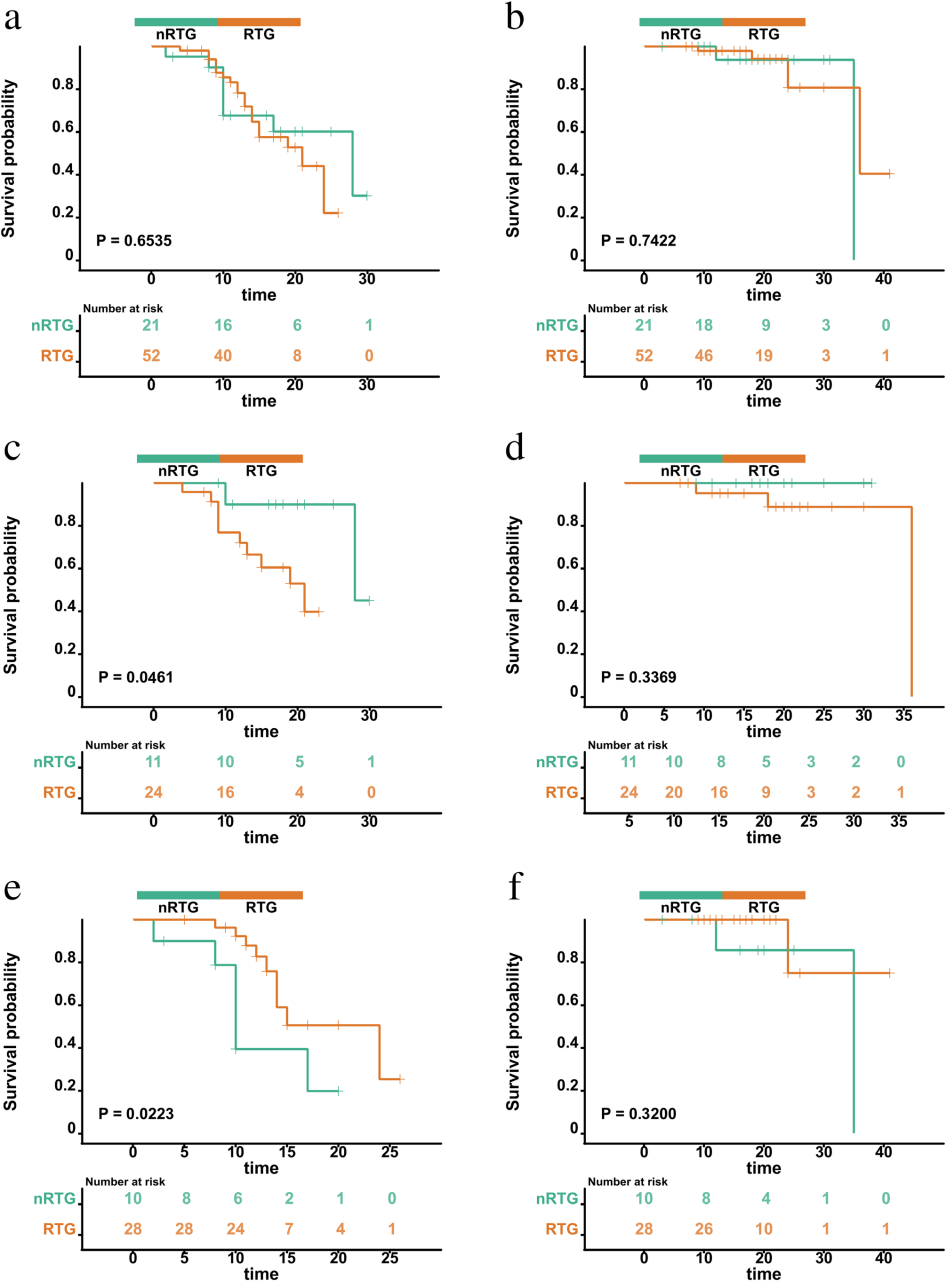
PFS and OS of patients in relation to radiotherapy (RT) status and PET/CT examination. PFS (a) and OS (b) comparison between the RTG and the nRTG. PFS (c) and OS (d) comparison between the RTG and the nRTG among patients who underwent PET/CT. PFS (e) and OS (f) comparison between the RTG and the nRTG among patients who did not undergo PET/CT.

Among patients who did not undergo PET/CT, the 1‐year PFS rates were 39.4% in the nRTG group and 82.7% in the RTG group. The 2‐year PFS rate in the RTG was 25.3%, while it was not observed in the nRTG (*p* = 0.022). The 1‐ and 2‐year OS rates were 85.7% versus 100.0% and 85.7% versus 75.0%, respectively (*p* = 0.320). In this subgroup, the RTG showed better PFS compared to the nRTG, but OS remained comparable between groups (Figure 2a–f).

Among patients who underwent PET/CT, the 1‐year PFS rates were 90.0% in the nRTG group and 72.1% in the RTG group. The 2‐year PFS rate in the nRTG was 90.0%, while it was not observed in the RTG (*p* = 0.046). The OS rates at 1 and 2 years were 100.0% versus 95.2% and 100.0% versus 88.9%, respectively (*p* = 0.337). In this subgroup, the RTG showed poorer PFS than the nRTG, but no significant OS benefit was observed.

**FIGURE 3:**
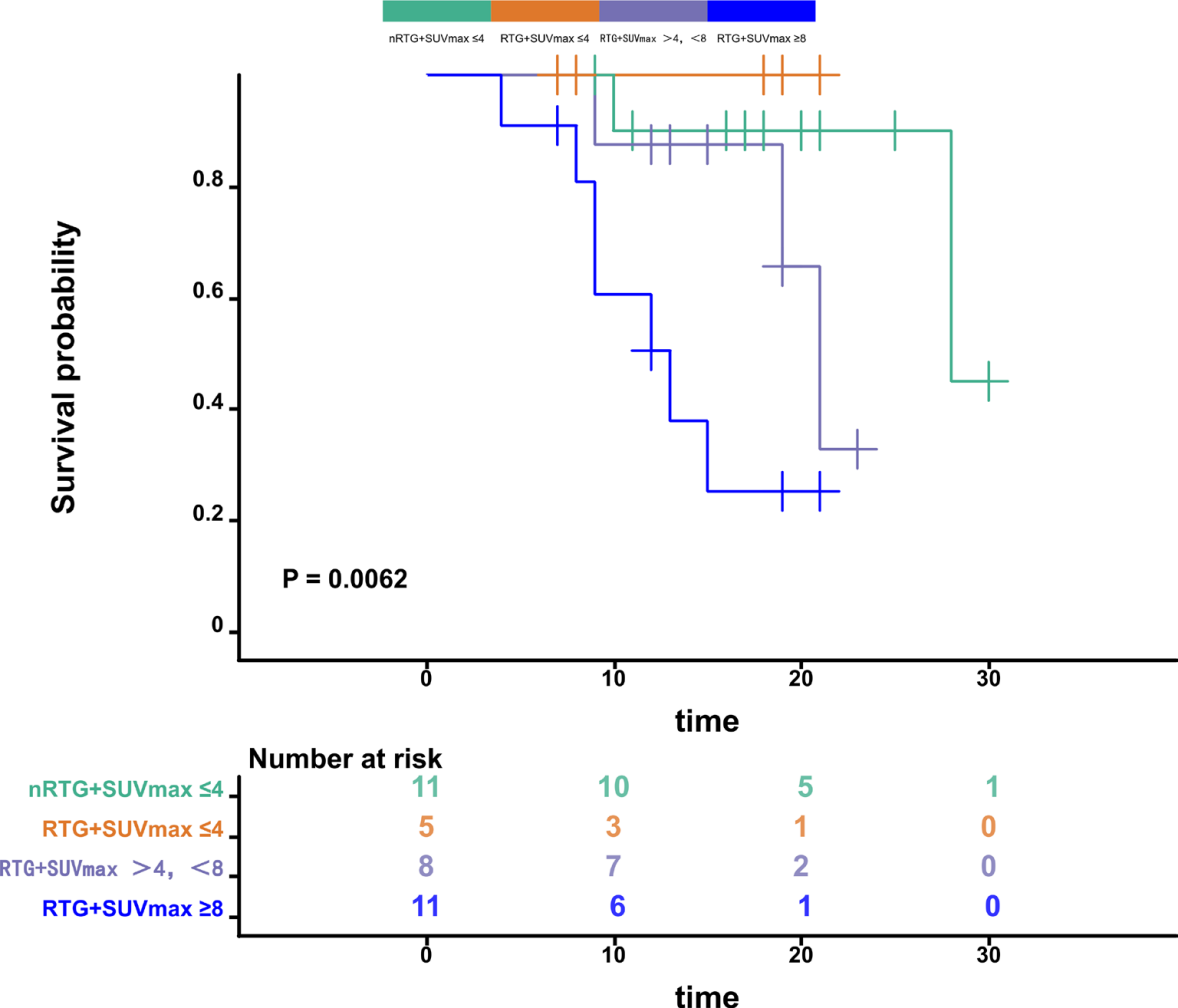
PFS of patients who underwent PET/CT stratified by radiotherapy (RT) status and SUV‐max values: nRTG + SUV‐max ≤ 4, RTG + SUV‐max ≤ 4, RTG + SUV‐max > 4 and < 8, and RTG + SUV‐max ≥ 8.

Further stratified analysis indicated no statistically significant difference in PFS between the nRTG and the RTG with SUV‐max ≤ 4 or 4–8. However, a significant difference in PFS between the RTG with SUV‐max ≥ 8 and the nRTG (*p* = 0.005) was observed (Figure 3).

### 3.3 Patterns of Disease Recurrence

As of the cut‐off date, progressive disease was observed in eight patients (38.1%) in the nRTG and 18 (34.6%) in the RTG. Local relapse occurred in seven patients (33.3%) in the nRTG group and in 14 patients (26.9%) in the RTG group. Distant metastases were reported in one patient (4.8%) in the nRTG and four patients (7.7%) in the RTG. Furthermore, five patients (9.6%) in the RTG experienced disease progression outside the radiation field.

### 3.4 Safety

In the RTG, Grade 5 radiation pneumonitis occurred in one patient (1.9%), while Grade 3/4 and Grade 1/2 radiation pneumonitis were observed in 2 (3.8%) and 21 patients (40.4%), respectively. Immune‐related pneumonitis was reported in six patients (11.5%) in the RTG, all grade 1/2. No immune‐related pneumonitis was observed in the nRTG. In the RTG, 1 patient (1.9%) developed Grade 3/4 esophagitis, and 14 patients (26.9%) developed Grade 1/2 esophagitis. The most common adverse reactions in both groups were hematologic toxicities, most of which were grade 1/2. Other frequently observed adverse reactions included radiation‐induced skin damage, fatigue, electrolyte imbalances, thyroid dysfunction, peripheral neuropathy associated with chemotherapy, and rash (Table 4).

**TABLE 4:** TRAEs between the RTG and nRTG.

TRAE	nRTG (*n* = 21)	RTG (*n* = 52)
Radiation pneumonitis	—	24 (46.2%)
G1/2	—	21 (40.4%)
G3/4	—	2 (3.8%)
G5	—	1 (1.9%)
Immune‐related pneumonitis	0 (0)	6 (11.5%)
G1/2	—	6 (11.5%)
G3/4	—	0 (0)
G1/2	—	0 (0)
Esophagitis	—	15 (28.8%)
G1/2	—	14 (26.9%)
G3/4	—	1 (1.9%)
Hematologic toxicity	6 (28.6%)	30 (57.7%)
G1/2	4 (19.0%)	18 (34.6%)
G3/4	2 (9.5%)	12 (23.1%)
Dermatitis	1 (4.8%)	11 (21.2%)
G1/2	1 (4.8%)	11 (21.2%)
G3/4	0 (0)	0 (0)

## 4 Discussion

At present, radical surgery remains the primary treatment for patients with Stage II and III NSCLC [3, 13, 14, 15]. However, postoperative recurrence remains a persistent clinical challenge [1, 16, 17, 18, 19]. Previous studies have demonstrated that the addition of perioperative chemotherapy to surgical intervention has only a limited effect on improving survival outcomes [4]. In recent years, immunotherapy targeting the PD‐1/PD‐L1 axis has gradually expanded from use in advanced NSCLC to early‐ and intermediate‐stage disease, achieving significant breakthroughs [5]. This development has marked the beginning of a new era of perioperative immunotherapy for patients with resectable NSCLC.

However, Stage II and III NSCLC is a highly heterogeneous disease, and in real‐world clinical settings, a considerable number of patients do not proceed to surgery following neoadjuvant therapy. In the CheckMate‐77T trial [8], 22.3% of patients did not undergo radical surgery. The reasons included disease progression (5.7%), patient refusal (4.8%), surgical cancelation by the surgeon (3.5%), inability to undergo surgery due to severe adverse events (3.1%), and other unspecified reasons (3.1%). Similarly, in the KEYNOTE‐671 trial [20], 17.9% of patients did not receive surgical treatment due to factors such as disease progression or severe cardiopulmonary conditions. In the CheckMate 816 trial [7], the reasons for not undergoing surgery following NACI included disease progression (6.7%), adverse events (1.1%), and other causes such as patient refusal, poor pulmonary function, or unresectable disease (7.8%).

The appropriate subsequent treatment strategy for patients who do not undergo surgery after NACI remains under discussion. Ding et al. [21] reported that PFS was 29.3 months in patients who received NACI followed by chemoradiotherapy (CRT), compared to 13.4 months in those who received immunotherapy after CRT (*p* < 0.001). Median OS was NR in patients who received NACI followed by CRT, but was 37.4 months in those who received immunotherapy after CRT (*p* = 0.004). Qi et al. [22] reported a median PFS of 25.9 months in patients upon treatment with concurrent chemoradiation and then followed by immunotherapy, while the median PFS was NR in patients who underwent NACI (either with surgery or radiotherapy) (HR: 2.91; 95% CI: 1.77–4.78; *p* < 0.001). Median OS was NR in either group, with 3‐year OS rates of 87.5% in the neoadjuvant group and 75.0% in the concurrent CRT plus immunotherapy group (*p* = 0.22).

The incidence of pneumonitis appears to be increased in patients who receive radiotherapy following immunotherapy. In a study by Shaverdian et al. [23], 41 patients with a history of immune‐related adverse events (irAEs) associated with immune checkpoint inhibitors who subsequently underwent thoracic radiotherapy were analyzed. Most irAEs were Grade 2 (*n* = 21) or Grade 3 (*n* = 19). Overall, 25 patients (61%) developed ≥ Grade 2 or higher radiation pneumonitis (RP) at a median of 4 months after radiotherapy and 11 months after irAE onset. At 3 months following RP onset, persistent symptoms were observed in 16 of 24 (67%) evaluable patients. Among those with previous ICI‐related pneumonitis (*n* = 6), five patients (83%) developed ≥ Grade 2 RP, including three cases of Grade 2 and two cases of Grade ≥ 3 disease.

Chen et al. [24] analyzed 96 patients who had previously received PD‐(L)1 inhibitor therapy and subsequently underwent thoracic radiotherapy. During follow‐up, 47 patients (48.96%) developed symptomatic treatment‐related pneumonitis, including 28 cases of Grade 2 and 19 cases of Grade ≥ 3, while six patients (6.25%) experienced fatal toxicity.

These findings indicate that patients receiving thoracic radiotherapy after immunotherapy face an elevated risk of radiation pneumonitis. As a result, both clinicians and patients may adopt a more cautious approach when selecting subsequent treatments. For example, in patients demonstrating favorable responses to NACI on PET/CT, thoracic radiotherapy may be omitted to reduce the risk of severe pneumonitis. Therefore, in this study, concern regarding pneumonitis was the primary reason most patients in the nonradiotherapy group declined radiotherapy.

In this retrospective study, the safety and efficacy of subsequent RT were compared to those of non‐RT patients without surgery after NACI. The findings indicated no significant difference in OS or PFS between the RTG and nRTG. The reason for this outcome is that our choices were biased. Most patients with a PR on CT or a PET/CT SUV‐max ≤ 4 chose to avoid radiotherapy, whereas all patients with PET/CT SUV‐max > 4 opted for radiotherapy as their primary subsequent treatment.

Among the patients who did not undergo PET/CT examination after receiving neoadjuvant chemotherapy, there was no selection bias. The research results show that the PFS of RTG is better than that of nRTG, indicating that radiotherapy has significant value for patients who did not undergo PET/CT. On the other hand, among those who underwent PET/CT after NACI, PFS was worse in the RTG than in the nRTG. Stratification by PET/CT SUV‐max revealed no significant difference in PFS between the nRTG + SUV‐max ≤ 4, RTG + SUV‐max ≤ 4, and RTG + SUV‐max > 4, < 8. However, among patients with SUV‐max ≤ 4, the nRTG survival appears acceptable from the survival curve, but it remains worse than RTG. Although some comparisons did not show a statistically significant difference, the survival curve trend suggested a survival advantage in the RTG. In this subgroup, no disease progression was observed in the RTG, while two cases occurred in the nRTG, including one local‐regional recurrence and one distant metastasis (liver). Patients with RTG + SUV‐max < 8 and RTG + SUV‐max ≥ 8 had poorer survival outcomes than those with nRTG + SUV‐max ≤ 4, possibly due to higher tumor metabolic activity in the RTG. A higher SUV value indicates a higher tumor burden and is associated with a greater likelihood of disease progression.

Overall, in clinical practice, for patients who have not undergone PET/CT after NACI, the advantages of radiotherapy are greater than for those who have not received it. For patients who undergo PET/CT examination after NACI, those with PET/CT‐SUVmax ≤ 4 without radiotherapy are also a treatment option. In patients with a PET/CT SUVmax ≥ 8, post‐NACI treatment response is considered suboptimal, reflecting relatively high residual tumor metabolic activity. These patients are therefore at increased risk of disease progression during subsequent management. In clinical practice, an aggressive, comprehensive treatment strategy with radiotherapy as the primary component should be actively considered for this patient population. These findings highlight the clinical value of PET/CT in guiding subsequent treatment decisions for patients who did not have surgery after NACI.

Yang et al. [25] reported the Grade 5 and Grade 3–4 pneumonitis incidence as 4.9% and 9.8%, respectively, in the group receiving induction ICIs plus chemotherapy before CRT, and 0% and 3.7%, respectively, in the group receiving consolidation ICIs after CRT. In this study, one patient (1.9%) in the RTG developed Grade 5 pneumonitis after chest radiotherapy and subsequently died. The pneumonitis was classified as radiation‐induced. No Grade 5 toxicity was reported in the nRTG. Our study has confirmed that the patient can be preliminarily screened using PET/CT after NACI. Patients without risk factors can be exempted from radiotherapy to avoid the occurrence of fatal radiation pneumonitis.

This study has several limitations, including its retrospective nature, single‐center setting, and relatively small sample size, particularly in the nRTG. A larger, prospective, randomized controlled trial is required to explore optimal treatment options after NACI. Of course, in the future, we will combine PET/CT with other biomarkers to jointly define the characteristics of patients who are more likely to benefit from nonsurgical treatment.

## 5 Conclusion

In conclusion, among patients who did not undergo surgery after NACI, no significant differences in OS or PFS were observed between the radiotherapy and nonradiotherapy groups. For patients who did not receive PET/CT evaluation after NACI, radiotherapy continues to play an important therapeutic role. In comparison, radiotherapy omission may be considered for patients with a PET/CT SUVmax ≤ 4 following NACI. However, patients with a PET/CT SUVmax ≥ 8 should receive a comprehensive treatment strategy that includes radiotherapy as the central component. These results suggest that PET/CT has meaningful clinical value in guiding subsequent treatment decisions. Future studies will expand the sample size and further investigate integrating PET/CT findings with additional biomarkers to optimize individualized treatment strategies.

## Author Contributions

All authors contributed to the study design, data collection, analysis, and manuscript revision. All authors read and approved the final manuscript.

## Funding

The authors have nothing to report.

## Ethics Statement

This study was conducted in accordance with the Declaration of Helsinki. Ethical approval was obtained from the Ethics Committee of National Cancer Center/National Clinical Research Center for Cancer/Cancer Hospital, Chinese Academy of Medical (Approval No. 25/205‐0205).

## Conflicts of Interest

The authors declare no conflicts of interest.

## Data Availability

The data that support the findings of this study are available on request from the corresponding author. The data are not publicly available due to privacy or ethical restrictions.
